# Genetic aberrations in DNA repair pathways: a cornerstone of precision oncology in prostate cancer

**DOI:** 10.1038/s41416-020-01114-x

**Published:** 2020-10-27

**Authors:** Rebeca Lozano, Elena Castro, Isabel M. Aragón, Ylenia Cendón, Carlo Cattrini, Pedro P. López-Casas, David Olmos

**Affiliations:** 1 Prostate Cancer Clinical Research Unit, Spanish National Cancer Research Centre (CNIO), Madrid, Spain; 2 Genitourinary Cancer Translational Research Group, The Institute of Biomedical Research in Málaga (IBIMA), Málaga, Spain; 3 UGCI Oncología Médica, Hospitales Universitarios Virgen de la Victoria y Regional de Málaga, Málaga, Spain; 4 Academic Unit of Medical Oncology, IRCCS San Martino Polyclinic Hospital, Genoa, Italy

**Keywords:** Molecular medicine, Predictive markers, Prognostic markers, Prostate cancer

## Abstract

Over the past years, several studies have demonstrated that defects in DNA damage response and repair (DDR) genes are present in a significant proportion of patients with prostate cancer. These alterations, particularly mutations in *BRCA2*, are known to be associated with an increased risk of developing prostate cancer and more aggressive forms of the disease. There is growing evidence that certain DDR gene aberrations confer sensitivity to poly-(ADP ribose) polymerase inhibitors and/or platinum chemotherapy, while other defects might identify cases that are more likely to benefit from immune checkpoint inhibition. The potential prognostic impact and relevance for treatment selection together with the decreasing costs and broader accessibility to next-generation sequencing have already resulted in the increased frequency of genetic profiling of prostate tumours. Remarkably, almost half of all DDR genetic defects can occur in the germline, and prostate cancer patients identified as mutation carriers, as well as their families, will require appropriate genetic counselling. In this review, we summarise the current knowledge regarding the biology and clinical implications of DDR defects in prostate cancer, and outline how this evidence is prompting a change in the treatment landscape of the disease.

## Background

The therapeutic landscape of metastatic castration-resistant prostate cancer (mCRPC) has rapidly evolved over the past 10 years as several agents have been shown to improve the overall survival (OS) of these patients.^1–7^ However, as no biomarker has yet been identified for treatment selection, prostate cancer has so far remained a disease treated with a “one-size-fits-all” approach.

The initiation of prostate cancer and the progression of the disease are driven by androgen receptor (AR) signalling, and androgen deprivation therapy (ADT) constitutes the backbone of systemic therapy for patients with advanced disease. However, insights into the biology of prostate cancer have shown that up to 60% of patients with advanced disease have clinically actionable molecular alterations in non-AR-related pathways.^8^ In particular, mutations in the genes encoding components of the DNA damage response (DDR; Box 1), such as *BRCA1* and *BRCA2*, are common in prostate cancer^8–13^ Such mutations reduce the ability to effectively repair single- and double-strand DNA damage, and thus compromise genomic integrity. This knowledge has resulted in a growing interest in biomarker-driven clinical trials. For example, patients harbouring *BRCA1*/*2* mutations have been shown to be more vulnerable to the action of poly-(ADP ribose) polymerase (PARP) inhibitors (PARPi), agents that prevent cells from repairing DNA damage. There are still several caveats regarding which DDR alterations may sensitise cells to PARP inhibition and when in the treatment sequence is the right time to use PARPi. However, with the recent approval of two of these compounds (olaparib and rucaparib) to treat advanced disease stages, prostate cancer has finally met precision oncology.

Here, we review the prevalence of DDR defects and the clinical implications of these alterations in both localised and advanced prostate cancer, before discussing data from the numerous clinical trials addressing the potential benefit of drugs targeting the DDR pathway in prostate cancer and the challenges physicians may face to incorporate PARPi to daily clinical practice.

**Fig. 1:**
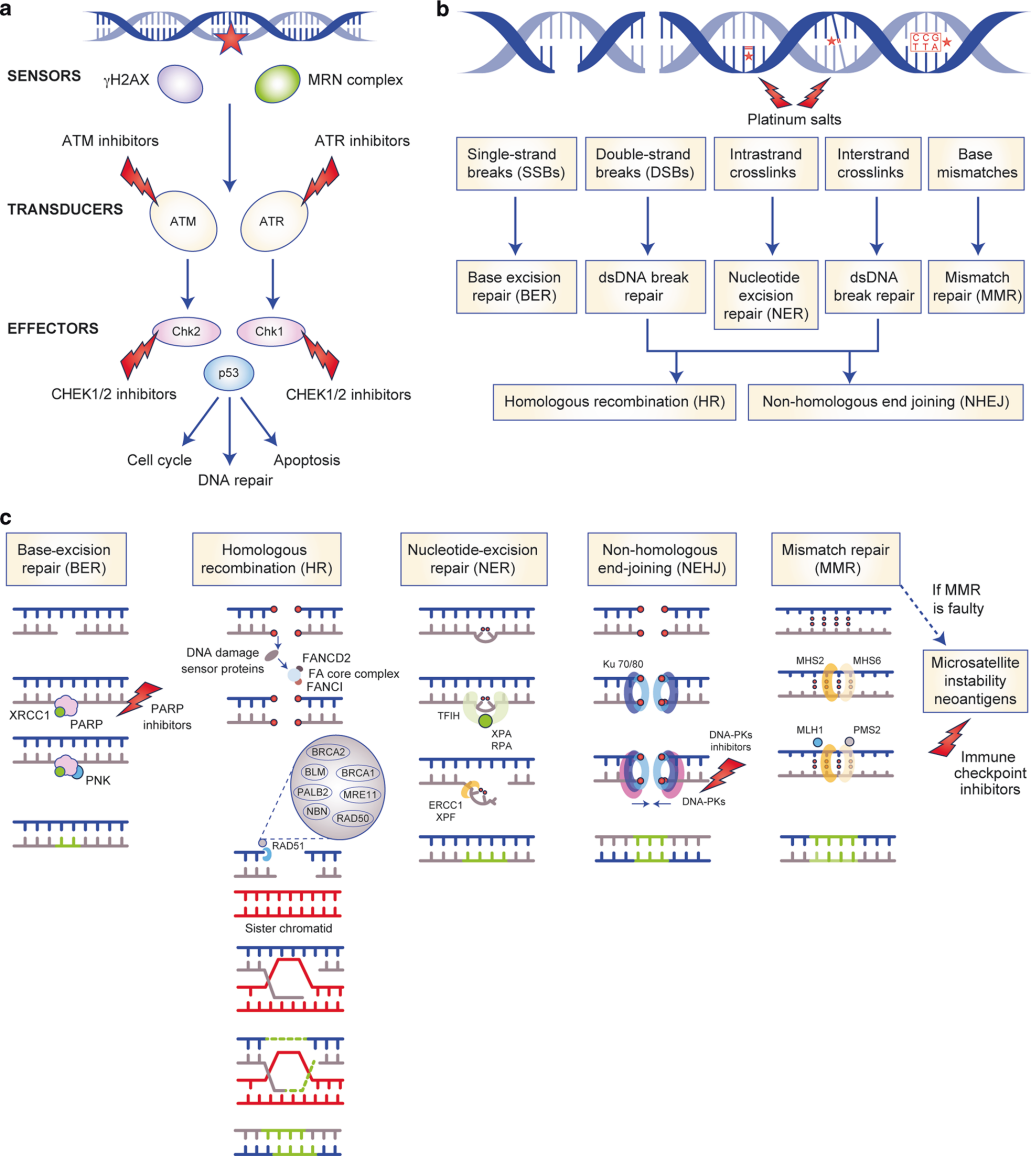
Representation of the different DNA damage response pathways. **a** DNA damage response is coordinated by different proteins which functions may be categorised in a simplified way as DNA damage sensors, transducers and effectors. DNA damage can be detected by γ-H2AX and MRN complex (sensors), which activate ATM and ATR. ATM and ATR are key signal transducers of downstream DDR pathways. They activate cell-cycle regulators CHK1 and CHK2 (encoded by *CHEK1* and *CHEK2*, respectively), which in turn signal downstream checkpoints that finally induce the activation of p53. This tumour suppressor determines if the cell initiates cell cycle, DNA repair mechanisms or undergoes apoptosis, depending on the DNA damage and/or DNA repair efficacy. **b** DNA-damaging agents may cause a range of different DNA lesions, which are repaired by a specific mechanism. ATR, ATM and CHK1/2 are kinases involved in the response to several types of DNA damage. Therefore, the inhibition of these kinases may be effective in certain tumours with DDR alterations or increase the activity or other agents causing DNA damage (i.e. platin salts). Similarly, DNA-PKc is a kinase involved in the NHEJ pathway, which inhibition by targeted drugs is also being tested in multiple tumours including prostate cancer. Platin salts (i.e. cisplatin or carboplatin) induce intra- and interstrand DNA crosslinks. These alterations require the coordinated action of HR, NHEJ and NER pathways to be repaired. When the function of genes involved in DNA repair pathway are altered (either in the somatic or in the germline), the cytotoxic effect of platinum chemotherapy could not be repaired, resulting in apoptotic cell death. PARP inhibitors interact with PARP, inhibiting its function. This result in the accumulation of single-strand breaks, which in turn leads to double-strand breaks (DSBs) formation. “Normal” cells have the ability to repair DSBs; however, these cannot be repaired in the presence of alterations in the function of genes involved in HR pathway (*BRCA1/2*, *PALB2*, FA family) (synthetic lethality). MMR deficiency leads to the accumulation of somatic mutations, and hence, more potential neoantigens. Higher somatic mutations and neoantigens have been correlated with better responses to immunotherapy.

Box 1 Human cells are continuously exposed to external (e.g. radiation) and internal (e.g. free radicals from our metabolism) noxious agents that can cause up to 10^5^ genetic lesions per cell every day, each of which has the potential to alter the DNA code.^81^ Maintaining genomic integrity is paramount to prevent the transmission of these errors onto daughter cells. In eukaryotic cells, such integrity is provided by elaborate surveillance systems and DNA repair mechanisms, each focusing on a specific category of DNA lesion, that respond to a harmful stimulus either by amending the damage or by initiating programmed cell death if the damage cannot be repaired.^82^ Thus, in concert with replication, transcription, recombination, chromatin remodelling and differentiation processes, DNA integrity is maintained.^83^

When DNA damage occurs, the cellular response is mediated by different proteins that can be grouped as sensors (i.e. γH2AX or MRN), transducers (i.e. ATM or ATR) or effectors (i.e. Chk1 or Chk2) of DNA damage (Fig. 1a). When damage is limited to one of the DNA strands (single-strand breaks, SSBs), different repair mechanisms can be activated, including base-excision repair (BER), SSB repair (SSBR), nucleotide-excision repair (NER) and mismatch repair (MMR). The BER pathway involves the removal of subtle modifications of DNA, such as oxidative lesions or small amounts of alkylation.^84^ PARP1 and PARP2 proteins are involved in detecting SSBs, formed either directly or as intermediates in BER, as well as in the coordination of the SSBR response. The NER pathway eliminates helix-distorting DNA damage or bulky DNA lesions caused by ultraviolet A light, a broad category of damage that affects one of the two DNA strands.^85^ Finally, MMR is a DNA repair system that recognises incorrectly paired nucleotides and erroneous insertions or deletions that also cause helix distortion^86^ and is important for maintaining genomic stability in regions with short, repetitive DNA sequences (i.e. microsatellites)*. MLH1*, *MSH2*, *MSH6* and *PMS2* are critical genes for the MMR (Fig. 1b).

Double-strand breaks (DSBs) are the most cytotoxic DNA lesions. The principal mechanisms to repair DSBs are the homologous recombination repair (HRR) and the non-homologous end-joining (NHEJ) pathways. The HRR pathway is a very complex, high-fidelity pathway that restores the original DNA code in an error-free mode but requires a sister chromatid as a template,^87^ and thus is restricted to the S and G2 phases of the cell cycle. Key mediators of HRR include, among many others, BRCA1, BRCA2, PALB2 and RAD51 proteins. HRR is also involved in the Fanconi anaemia pathway, which removes DNA interstrand crosslinks involved in the DSBs.^88^ The NHEJ repair system is active throughout the cell cycle and repairs DSBs by religation of the DNA ends without using a guidance template—thus, this process is error-prone and might introduce new mutations^89^ (Fig. 1b). The balance between both pathways is essential for the maintenance of genome stability.

Despite the specificity in repairing different lesion types, some overlap and cooperation of different repair mechanisms have also been described—particularly for repairing the more complex lesions.

## Prevalence of alterations in DDR genes in prostate cancer

The high prevalence of genomic alterations that involve DDR genes in prostate cancer has been recognised over the past 5 years.^8–14^ In 2015, The Cancer Genome Atlas (TCGA) published the molecular analysis of 333 primary prostate tumours, revealing that 19% of them harboured alterations in different DDR genes, including *BRCA2*, *BRCA1*, *ATM*, *CDK12*, *FANCD2* or *RAD51C*.^12^ Even though *BRCA2* was reported as the most commonly altered gene, all six cases with germline *BRCA2* mutations presented with the same variant, p.K3326* (the pathogenic significance of which is unclear). At the same time, a report of the International Stand Up to Cancer/American Association for Cancer Research Prostate Cancer/Prostate Cancer Foundation Team (SU2C-PCF) identified genomic alterations affecting DDR genes in 23% of the 150 metastatic biopsy samples analysed. *BRCA2* was altered in 13% of samples, followed by *ATM* (7.3%), *MSH2* (2%) and *BRCA1, FANCA, MLH1, RAD51B* and *RAD51C* (all with a prevalence of 0.3%).^8^ A larger series that included samples from the aforementioned studies identified DDR defects in 10% and 27% of the primary and metastatic samples, respectively,^14^ in line with the observation that DDR alterations are associated with high histology grade and metastatic prostate cancer.^15–18^ Besides, the prevalence of DDR alterations across different studies may vary depending on the number of genes analysed, the technique used and the clinico-pathological features of the tumours included. The larger series of prostate cancer samples screened for DDR defects has been provided by the PROfound study, a Phase 3 study addressing the benefit of the PARPi olaparib in mCRPC, that successfully screened 2792 biopsies for aberrations in 15 DDR genes involved in the homologous recombination repair (HRR) pathway.^19^ Such alterations were identified in 28% of the samples analysed,^20^ with a similar frequency in primary tumours (27%) and in biopsy samples from metastatic sites (32%), suggesting that HRR alterations are probably early events in the evolution of aggressive prostate tumours. *BRCA2* (8.7%), *CDK12* (6.3%), *ATM* (5.9%), *CHEK2* (1.2%) and *BRCA1* (1%) were the most commonly altered genes. Co-occurring aberrations in two or more DDR genes were found in 2.2% of cases, although it is unclear whether this is associated to increased sensitivity to PARPi. The prevalence of mismatch repair (MMR) defects in prostate cancer was established in 2018 in a large series with 1033 patients. In this study, Abida et al.,^21^ identified a MMR deficiency in 3.1% of cases, and confirmed *MSH2* as the most commonly altered MMR gene in prostate cancer (Table 1).

**Table 1:** Prevalence of DDR alterations in localised and advanced prostate cancer.

Gene	Localised disease^12,21^	Advanced disease^8–10,13,21,32^
*BRCA2*	3%	13.3%
*BRCA1*	1%	1.9%
*ATM*	4%	7.3%
*CHEK2*	0%	1.9%
*CDK12*	2%	6.9%
*PALB2*	–	0.4%
*RAD51C*	3%	0.14%
*RAD51D*	0%	0.4%
*FANCD2*	6%	0.7%
*MLH1*^a^	0.6%	1.3%
*MSH2*^a^	1.2%	2.7%
*MSH6*^a^	1.4%	2%

^a^The frequency of mismatch repair deficiency in a paper, including both localised and advance disease reported by Abida et al.^21^ was 3.1%.

A remarkable finding from the SU2C-PCF study was that 8% of the mCRPC patients harboured a germline DDR mutation—almost half of all the reported DDR aberrations.^8^ This study was the first to suggest that germline variants in DDR genes associated with an increased risk of cancer were present in metastatic prostate cancer at a prevalence higher than previously acknowledged. A first confirmation of these findings was provided by a retrospective multicentre study that pooled data on germline DNA variants from 692 patients with metastatic prostate cancer from different series (including the aforementioned SU2C-PCF study). In this analysis, 11.8% of the patients were identified as carriers of a deleterious germline mutation in at least one of the 20 DDR genes associated with cancer-predisposition syndromes that were analysed.^9^ A slightly lower prevalence (7.4%) of mutations in the same genes was reported in PROREPAIR-B, a prospective study^13^ that screened 419 unselected patients with mCRPC from Spain. This variation in prevalence is likely to be due to the different genetic background of both populations as the series reported by Pritchard et al.^9^ included a higher prevalence of the Ashkenazi founder mutations *BRCA1* c.5266dupC and *BRCA2* c.5946delT and the Eastern European founder mutation *CHEK2* p.1100del than the prospective Spanish study. Nonetheless, *BRCA2* was the most commonly mutated gene in both series (5.3%^9^ and 3.3%,^13^ respectively).

It is noteworthy that most of the studies analysing the presence of DDR defects in tumours do not distinguish the germline or somatic origin of the variants identified. A 2019 analysis of over 17,000 tumours, including 1042 prostate cancers, identified *BRCA2* aberrations in 92 cases. Unlike for the other cancer types included, the proportion of prostate tumours with germline and somatic *BRCA2* mutations was similar and approximately half of the *BRCA2* alterations identified through tumour profiling are already present in the germline. Furthermore, the authors describe prostate tumours as the only *BRCA2*-mutated prostate cancers with a similar rate of biallelic loss (70%) in cases with germline and somatic alterations.^22^ A lower ratio of germline:somatic alterations have been reported for components of the MMR pathway in prostate cancer, as less than a quarter (22%) of the tumours identified by Abida et al.^21^ as MMR deficient harboured a germline mutation. This is in line with previous reports that found germline mutations significantly less prevalent in the prostate than in other cancer types with MMR defects such as the colorectal or urothelial ones.^23^

## Clinical implications of DDR gene alterations in prostate cancer

Due to the high prevalence of germline mutations in DDR genes, the National Comprehensive Cancer Network (NCCN) has recommended germline testing for all men with high-risk localised prostate cancer and in those with metastatic disease.^24^ The development of PARPi to treat prostate cancer has made genomic testing appealing in this setting. Therefore, the number of prostate cancer patients with tumours classified as DDR deficient is likely to increase in the near future. However, the clinical relevance of germline and somatic defects in DDR genes remains largely unclear at present—the exception being germline *BRCA2* mutations, which have been shown to be an independent prognostic factor for prostate cancer outcomes in different settings.^13,15,25,26^

### For non-metastatic prostate cancer patients

A range of management options, including active surveillance, radical prostatectomy and/or radiotherapy with or without hormonal deprivation, is currently available to treat patients with localised prostate cancer. Data regarding the implications of germline and somatic DDR defects in early prostate cancer come from retrospective analyses, most of them focused on germline *BRCA* mutation carriers.

A report on the outcomes of 1211 men undergoing active surveillance, including 11 *BRCA1*, 11 *BRCA2* and 5 *ATM* germline carriers, has shown that *BRCA2* carriers are more likely to undergo a tumour grade re-classification in subsequent biopsies. In this series, the incidence of tumour staging upgrades at 2, 5 and 10 years was 27%, 50% and 78% in *BRCA2* carriers compared with 10%, 22% and 40% in non-carriers (*P* = 0.001).^26^ These results indicate that active surveillance might not be an appropriate management for germline *BRCA2* carriers with tumours classified as “low risk”.

Likewise, no conclusive data are available regarding the potential use of alterations in *BRCA2* or other DDR genes for selecting between curative treatment options (radical prostatectomy or radiotherapy). The only existing evidence comes from a retrospective study that analysed the outcomes of 1302 patients, including 18 *BRCA1* and 49 *BRCA2* carriers, with localised disease. In this series, *BRCA1/2* carriers developed metastasis significantly earlier than non-carriers after radical treatment. Metastasis-free survival rates for those surgically treated were 89% and 67% in *BRCA1/2* carriers compared with 97% and 91% in non-carriers at 5 and 10 years, respectively (*P* = 0.024). The difference was even greater for patients treated with radiotherapy, as only 57% and 39% of *BRCA1/2* carriers were free from metastasis at 5 and 10 years, respectively, compared with 91% and 80% of non-carriers (*P* < 0.001).^25^ A direct comparison of the two groups could not be performed as patients treated with radiotherapy (both carriers and non-carriers) presented with more advanced disease than those who were surgically treated. Therefore, it is not possible to conclude from this study which of the treatment options—surgery or radiotherapy—would be best for *BRCA* carriers.

### For patients with metastatic hormone-sensitive prostate cancer and CRPC

The clinical implications of DDR defects in advanced prostate cancer remain unclear as the available evidence is somehow conflicting. To date, data on the prognosis of advanced prostate cancer patients with DDR gene aberrations and the response to therapies are limited. For metastatic hormone-sensitive prostate cancer (mHSPC), at least two studies in mCRPC patients have provided retrospective data on time-to-castration resistance (TTCR) from initiation of continuous ADT. Annala et al.^27^ reported that patients with germline mutations in HRR genes presented a significantly shorter TTCR than non-carriers (11.8 vs. 19.0 months, *P* = 0.031). Similarly, Castro et al.^13^ showed that germline *BRCA2* mutation carriers achieve mCRPC status earlier than non-carriers (13.2 vs. 28 months, *P* = 0.05). The only prospective data available to date has been reported by Vandekerkhove et al.^28^ This group analysed the outcomes of 53 patients with de novo mHSPC, and reported a significantly shorter TTCR in the 11 cases with somatic and/or germline DDR alterations compared with those without such aberrations (7.3 months vs. not reached, *P* = 0.01). However, this difference did not remain significant in the multivariable analysis.^28^

Three retrospective studies assessed the role of germline DDR mutations in the outcomes of patients with mCRPC treated with AR signalling inhibitors (ARSi) and taxanes. In the first report, Annala et al.^29^ analysed 176 mCRPC patients, including 22 germline carriers (16 *BRCA2*). They found that carriers treated with ARSi as first-line therapy had shorter progression-free survival (PFS) than non-carriers (3.3 vs. 6.2 months, *P* = 0.01), despite apparent prolonged responses to ARSi in some carriers. Importantly, almost all carriers in this series had a circulating tumour DNA (ctDNA) fraction relative to total cell-free DNA of >30%, which correlates strongly with disease burden, poor responses and shorter OS.^27^ Therefore, it is questionable whether the poor clinical outcomes of these patients are related solely to their germline status, or potentially also affected by other confounding factors, such as tumour burden and a high ctDNA. In a second report, the clinical outcomes of 172 mCRPC patients, including 22 germline carriers (five *BRCA2*) who were treated with ARSi, were analysed by Antonarakis et al.^30^ In this study, carriers showed a trend to longer PFS than non-carriers (15 vs. 10.8 months, *P* = 0.090). Interestingly, those patients who received chemotherapy before ARSi had shorter PFS and cause-specific survival (CSS), suggesting that treatment sequence could be important in this setting. The third report^31^ analysed the outcomes of 390 mCRPC patients included in the series published previously by Pritchard et al.^9^ describing the prevalence of germline DDR alterations. This study included 60 germline DDR mutation carriers (37 *BRCA2*). No significant differences in the responses to the administered therapies or in OS were observed between carriers and non-carriers. However, it should be noted that, unlike in the other studies, up to 47% of carriers in this series had received platinum salts and/or PARPi, which could have a confounding effect on the outcomes.

PROREPAIR-B^13^ was the first prospective study designed to evaluate the impact of germline DDR mutations on the outcome(s) of mCRPC patients. Out of a total of 419 patients analysed, 68 were found to carry a germline mutation (14 *BRCA2*). Remarkably, in this cohort, in which none of the carriers received PARPi or platinum salts, CSS was halved in *BRCA2* carriers compared with non-carriers (17.4 vs. 33.2 months, *P* = 0.027). The multivariate analyses identified germline *BRCA2* mutations as an independent prognostic factor for CSS in this setting (HR [hazard ratio] 2.11; 95% CI [confidence interval] 1.06–4.18). The differences in CSS were not significant when the outcomes of patients with *BRCA2* mutations were analysed together with those harbouring germline *ATM* or *BRCA1* mutations (23.3 vs. 33.2 months, *P* = 0.264); neither were they significant when carriers of any germline mutation in the 107 DDR genes studied in PROREPAIR-B were compared with non-carriers (28.6 vs. 33.1 months, *P* = 0.646). Germline mutations in *BRCA2* have clearly a deleterious impact on the prognosis of mCRPC patients, while germline mutations in other DDR genes may not affect patients’ survival. Further studies are needed to establish the clinical significance of inherited mutations in less frequently altered genes.

Exploratory analyses in PROREPAIR-B^13^ showed that germline *BRCA2* carriers had worse CSS than non-carriers when they received first-line taxane therapy for mCRPC (10.7 vs. 28.4 months, *P* < 0.001). By contrast, there were no significant differences in CSS between germline *BRCA2* carriers and non-carriers treated initially with ARSi for mCRPC (24.0 vs. 31.2 months, *P* = 0.901). CSS outcomes were similar for non-carriers treated with either a first-line ARSi or taxane. Despite further validation of these results is required, germline *BRCA2* status may be relevant for tailoring the treatment sequence in mCPRC, and mHSPC.

In the past year, relevant data on the clinical implication of *CDK12* and MMR alterations have been reported. *CDK12* defects are present in 6% of mCRPC^19^ patients and are often biallelic.^32^ *CDK12* is a kinase involved in HRR but is also key to maintain genomic stability.^32^ Four retrospective series have demonstrated the clinical aggressiveness of *CDK12*-inactivated prostate cancer tumours, which often present Gleason grade groups 4 and 5 and metastases at diagnosis. These patients had a rapid progression to castration resistance from ADT initiation and responses to abiraterone and enzalutamide were poor.^16–18,33^ Importantly, one of these studies has suggested that *CDK12* alterations may confer poorer outcomes than somatic defects in *BRCA1/2*, *ATM* or *TP53*.^16^ MMR have also been associated with aggressive features and more advanced disease at diagnosis.^34,35^ However, conflicting results have been reported on the clinical outcomes of MMR-deficient prostate tumours.^34^ While a study suggests favourable responses to ADT, others have found that MMR-deficient patients develop castration-resistant disease earlier than the MMR-proficient ones.^35,36^ These differences in outcomes between studies may be related to the limited number of patients included and the different assays used to detect these alterations (targeted sequencing vs. immunohistochemistry). Further studies are needed to completely elucidate the clinical implications of these and other DDR alterations in prostate cancer.

## Targeting DDR genes in prostate cancer

The relatively high prevalence of alterations in DDR genes in patients with advanced prostate cancer provides a unique opportunity to take advantage of these defects by using different therapeutic strategies, including synthetic lethality.

### Platinum-based chemotherapy

Platinum-based chemotherapy causes DNA crosslinks that cannot be easily repaired when the HRR pathway is impaired, leading to cell death (Box 1). This strategy has proven successful in treating breast^37,38^ and ovarian^39^ cancers with pathogenic mutations in *BRCA1* or *BRCA2*. Although platinum salts are not a standard of care option in prostate cancer patients, their use is recommended in cases of neuroendocrine differentiation.^40,41^ In the DDR context, several retrospective studies suggest that *BRCA2*-mutated prostate cancer patients might benefit from this treatment approach.^42–45^ The largest of these analyses included 141 men with mCRPC treated with carboplatin and docetaxel at the Dana Farber Cancer Institute between 2001 and 2015, and reported a benefit from this combination for patients with germline *BRCA2* mutations.^43^ Six out of the eight *BRCA2* carriers (75%) showed a >50% decline in the levels of prostate-specific antigen (PSA) at 12 weeks, compared with 23 of 133 of non-carriers (17%) (*P* = 0.001). A >50% PSA decline was associated with longer survival (18.9 months in *BRCA2* carriers vs. 9.5 months in non-carriers). A second study also evaluated the efficacy of platinum-based chemotherapy after progression to taxanes in 109 mCRPC patients.^45^ A PSA decline ≥50% was more frequent in patients with DDR alterations compared with DDR-proficient patients (50% vs. 13%, *P* = 0.006). This analysis included a subset of patients with DDR aberrations who received platinum chemotherapy after progression on PARPi (*n* = 8) with clinical benefit observed in a third of them. None of the two patients with ATM mutations responded to a platinum, regardless of prior treatment with PARPi. Several studies are ongoing to validate these results and to establish the role of platinum-based chemotherapy for DDR-deficient prostate tumours in different scenarios (Table 2).

**Table 2:** Ongoing clinical trials assessing PARPi, platinum chemotherapy or other drugs impairing the DDR pathway in prostate cancer.

Clinical trial	Phase	Drug	Study population	DDR defects screening	Strategy	Primary endpoint
NCT02861573 (KEYNOTE-365)	1	Olaparib	mCRPC	✗	Cohort A: pembrolizumab + olaparib in post-docetaxel setting	PSA50 response rate
NCT03874884 (LuPARP)	1	Olaparib	mCRPC	✗	Olaparib + 177 Lu-PSMA therapy after chemotherapy and ARSI	MTD, DLT and RP2D
NCT03317392	1/2	Olaparib	mCRPC	✗	Ra223 ± olaparib in mCRPC patients with bone metastases	MTD of combination and rPFS
NCT03787680 (TRAP trial)	2	Olaparib	mCRPC	✓	Olaparib + ATR inhibitor (AZD6738) in second-line setting	Response rate
NCT03432897 (BrUOG 337)	2	Olaparib	Locally advanced Prostate cancer	✓	Olaparib prior to radical prostatectomy	PSA response rate
NCT03012321	2	Olaparib	mCRPC	✓	Olaparib ± abiraterone/prednisone in first-line setting	PFS
NCT03434158 (IMANOL)	2	Olaparib	mCRPC	✗	Olaparib for patients who are responding after docetaxel chemotherapy	rPFS
NCT03570476	2	Olaparib	Localised PCa	✓	Olaparib before radical prostatectomy	pCR rate
NCT03047135	2	Olaparib	Biochemically recurrent High-risk PCa	✓	Olaparib in biochemically recurrent prostate cancer	PSA response rate
NCT03516812	2	Olaparib	mCRPC	✓	Olaparib + testosterone enanthate in post-abiraterone/enzalutamide setting	PSA50 response rate
NCT02893917	2	Olaparib	mCRPC	✗	Olaparib ± cediranib in second-line setting	rPFS
NCT03810105	2	Olaparib	Biochemically recurrent nmHSPC	✓	Olaparib + durvalumab in biochemically recurrent PCa	Undetectable PSA
NCT03732820	3	Olaparib	mCRPC	✗	Abiraterone/prednisone ± olaparib in first-line setting	rPFS
NCT03834519 (KEYLINK-010)	3	Olaparib	mCRPC	✗	Olaparib + pembrolizumab vs. ARSI after chemotherapy and ARSI	OS and rPFS
NCT03076203 (NiraRad)	1	Niraparib	mCRPC	✗	Niraparib + radium-223	MTD
NCT03431350 (QUEST)	1/2	Niraparib	mCRPC	✓	Niraparib + sbiraterone/prednisone or JNJ-63723283 in post-ARSI setting	Incidence of toxicities and ORR
NCT04037254	1/2	Niraparib	High-risk localised PCa	✗	Niraparib + RT + ADT vs. RT + ADT (Phase 2)	DF state at 24 months
NCT02854436 (Galahad)	2	Niraparib	mCRPC	✗	Niraparib in post-docetaxel and post-ARSI setting	ORR
NCT04030559	2	Niraparib	High-risk localised PCa	✓	Niraparib before radical prostatectomy	pRR
NCT03748641 (MAGNITUDE)	3	Niraparib	mCRPC	✓	Abiraterone/prednisone ± niraparib in first-line setting	rPFS
NCT03840200	1b	Rucaparib	Advanced breast, ovarian and PCa	✗	Rucaparib + ipatasertib in post-ARSI setting (part 2)	AE, DLT, PSA response rate
NCT03572478	1/2	Rucaparib	mCRPC and endometrial cancer	✗	Rucaparib vs. rucaparib + nivolumab vs. nivolumab (Phase 2b randomised cohort)	DLT
NCT03413995 (TRIUMPH)	2	Rucaparib	mHSPC	✓	Rucaparib without ADT (mHSPC without large lymph nodes and visceral disease)	PSA response rate
NCT02952534 (TRITON2)	2	Rucaparib	mCRPC	✓	Rucaparib in post-docetaxel and post-ARSI setting	ORR
NCT03533946 (ROAR)	2	Rucaparib	nmHSPC	✓	Rucaparib in nmHSPC with PSADT < 10 months	PSA50 response rate
NCT03338790 (CheckMate 9KD)	2	Rucaparib	mCRPC	✗	Nivolumab + rucaparib or docetaxel or enzalutamide	ORR
NCT03442556	2	Rucaparib	mCRPC	✓	Rucaparib for patients who are responding after docetaxel plus carboplatino	rPFS
NCT02975934 (TRITON3)	3	Rucaparib	mCRPC	✓	Rucaparib vs. abiraterone/enzalutamide/docetaxel in second-line setting	rPFS
NCT04019327	1/2	Talazoparib	mCRPC	✓	Intermittent talazoparib and temozolomide in DDR-negative patients	AE (Phase 1), ORR (Phase 2)
NCT04052204	1/2	Talazoparib	mCRPC and mSCCHN	✓	Avelumab + NKTR-214 ± talazoparib	DLT, ORR
NCT03330405 (Javelin Parp Medley)	2	Talazoparib	Locally advanced or metastatic tumours	✗	Avelumab plus talazoparib in advanced solid tumours	DLT
NCT03148795 (TALAPRO-1)	2	Talazoparib	mCRPC	✓	Talazoparib in post-docetaxel and post-abiraterone/enzalutamide setting	ORR
NCT03395197 (TALAPRO-2)	3	Talazoparib	mCRPC	✓	Enzalutamide ± talazaparib in first-line setting	rPFS
NCT04038502 (BRACeD)	2	Carboplatin	mCRPC	✓	Carboplatin (1 L) followed by docetaxel (2 L) vs. opposite sequence	PFS
NCT03652493 (PRO-CARBO)	2	Carboplatin	mCRPC	✓	Carboplatin after chemotherapy and ARSI	RR, PSA response rate
NCT02311764 (PRO-PLAT)	2	Carboplatin	mCRPC	✓	Carboplatin after chemotherapy and ARSI	RR (radiographic and PSA)
NCT02955082 (BARCODE2)	2	Carboplatin	mCRPC	✓	Carboplatin after chemotherapy and ARSI	RR (radiographic and PSA)
NCT02598895 and NCT02985021	2	Carboplatin	mCRPC	✓	Carboplatin and docetaxel in post-chemotherapy and post-ARSI setting	PSA50 response rate
NCT03442556 (PLATI-PARP)	2	Carboplatin	mCRPC	✓	Carboplatin and docetaxel followed by rucaparib maintenance after chemotherapy and ARSI	rPFS
NCT03517969	2	M6620	mCRPC	✗	M6620 (ATR inhibitor) + carboplatin vs. docetaxel + carboplatin in third-line setting	ORR
NCT03787680 (TRAP trial)	2	AZD6738	mCRPC	✓	AZD6738 (ATR inhibitor) + olaparib in second-line setting	ORR
NCT02203513	2	LY2606368	Breast, ovarian and PCa	✓	LY2606368 (Chk1/2 inhibitor) in advanced prostate cancer	ORR

*DDR* DNA damage repair, *PCa* prostate cancer, *mCRPC* metastatic castration-resistant prostate cancer, *177 Lu-PSA* lutetium-prostate-specific membrane antigen, *DLT* dose-limiting toxicities, *RP2D* recommended Phase 2 dose, *mHSPC* metastatic hormone-sensitive prostate cancer, *nmHSPC* non-metastatic hormone-sensitive prostate cancer, *Ra223* Radium-223, *ARSI* androgen receptor signalling inhibitor (abiraterone, enzalutamide), *ADT* androgen deprivation therapy, *PSADT* PSA doubling time, *PSA50 response* 50% reduction in PSA levels from baseline, *MTD* maximum tolerated dose, *rPFS* radiographic progression-free survival, *PFS* progression-free survival, *pCR* pathological complete response, *RT* radiotherapy, *DF* disease free, *ORR* overall response rate, *pRR* pathological response rate, *AE* adverse events, *mSCCHN* metastatic squamous cell carcinoma of the head and neck, *1* *L* first-line treatment, *2* *L* second-line treatment.

### PARP inhibitors and synthetic lethality

Another strategy to treat DDR-deficient tumours is to exploit the synthetic lethal interaction between the inhibition of PARP and the impairment of HRR already present in some tumours. This interaction is thought to result from the PARPi-induced increase in the number of double-strand break (DSBs) or collapsed replication forks, which are lethal in HRR-deficient cells^46^ (Box 1). Several PARPi, differing in their ability to bind PARP and to trap PARP–DNA complexes,^47^ are at various stages of clinical development (Table 2). Olaparib was the first-in-class drug to be granted approval in 2014 for the treatment of *BRCA*-deficient ovarian cancer.^48^ The first-in-man clinical trial of olaparib in a population of patients with advanced solid tumours enriched for germline *BRCA1* and *BRCA2* mutations included three mCRPC patients, one of whom benefited from treatment with the drug for over 2 years.^49,50^ Small numbers of mCRPC patients with germline *BRCA* mutations were also enrolled in Phase 1 trials of other PARPi, such as talazoparib^51^ or niraparib as single agents,^52^ showing promising signs of antitumour activity in these patients with a good safety profile.

### TOPARP-A and TOPARP-B

In the TOPARP-A Phase 2 trial,^53^ 50 men with heavily pretreated mCRPC received olaparib 400 mg twice a day. Fourteen out of 16 patients (88%) who harboured a DDR defect (somatic or germline) achieved clinical benefit (including radiological responses, a decrease in PSA levels and/or a decrease in circulating tumour cell count) and durable responses. All seven patients with *BRCA2* defects, but also some with *BRCA1*, *ATM*, *PALB2* and *FANCA* defects, among others, responded. Subsequently, the TOPARP-B Phase 2 trial^54^ enrolled 98 mCRPC patients with DDR alterations. Half of them received 300 mg twice daily and the other half received 400 mg twice daily. Radiographic and PSA responses were observed in 39% and 16% of patients in the 300 mg twice daily cohort and in 54% and 24% of patients in the 400 mg twice daily cohort, respectively. Despite the increased responses and prolonged benefit in the 400 mg arm, 37% of these patients required a dose reduction to 300 mg twice daily due to toxicity. The study included 30 men with *BRCA1/2* mutations, of whom 52% and 77% achieved a radiographic or PSA response, respectively, as compared with 5% and 11.3% of men with other DDR defects.

### PROfound

PROfound^19^ is the first randomised Phase 3 biomarker-driven clinical trial in mCRPC patients. In this trial, DDR-deficient mCRPC patients who progressed on prior ARSi were randomised 2:1 to receive olaparib 300 mg twice daily or the alternative ARSi according to physician’s choice. Patients with a DDR alteration were stratified into two cohorts based on the previous evidence of activity: cohort A with *BRCA1*, *BRCA2* and *ATM* alterations; and cohort B, which included alterations in *BARD1*, *BRIP1*, *CDK12*, *CHEK1*, *CHEK2*, *FANCL*, *PALB2*, *PPP2R2A*, *RAD51B*, *RAD51C*, *RAD51D* and *RAD54L*. The primary endpoint was radiographic PFS (rPFS) benefit in cohort A. Crossover to olaparib was allowed upon progression. A total of 245 and 142 patients were included in cohorts A and B, respectively (65.6% had previously received taxane). The study met its primary endpoint and showed a statistically significant benefit in rPFS in patients included in cohort A, with a median rPFS of 7.4 months in men treated with olaparib vs. 3.5 months in those who received abiraterone or enzalutamide (*P* < 0.001; HR 0.34, 95% CI 0.25–0.47). In an exploratory analysis that looked at the gene-by-gene effect on rPFS, patients with *BRCA2* aberrations seemed to benefit the most from olaparib. The final analysis of overall survival has been already reported,^55^ showing a clear benefit in patients with BRCA1, BRCA2 and ATM mutations (cohort A), with a median OS of 19.1 months in the olaparib arm compared with 14.7 months with ARSi (HR 0.69, *p* = 0.02), despite 66% of patients in the control arm crossing over to olaparib at radiographic progression. Median OS in the overall population (cohorts A and B) was 17.3 vs 14.0 months (HR 0.79) with olaparib and hormonal treatment, respectively.

### TRITON2 and GALAHAD

The preliminary results of two Phase 2 trials (TRITON2 and GALAHAD) evaluating the efficacy of other two PARPi (rucaparib and niraparib, respectively) in heavily pretreated mCRPC patients have also been reported.^56,57^ Both studies have enrolled patients with DDR defects, although the assays and the gene panels used to screen patients and decide their eligibility are different (Table 3). Patients with alterations in *ATM*, *BARD1*, *BRCA1*, *BRCA2*, *BRIP1*, *CDK12*, *CHEK2*, *FANCA*, *NBN*, *PALB2*, *RAD51*, *RAD51B*, *RAD51C*, *RAD51D* or *RAD54L* detected in tissue, or *ATM, BRCA1* or *BRCA2* in plasma, are eligible for the TRITON2 study, while GALAHAD is only enrolling men with biallelic tumour alterations in *ATM*, *BRCA1*, *BRCA2*, *BRIP1*, *CHEK2*, *FANCA*, *HDAC2* or *PALB2* detected in circulating free DNA. Forty-four per cent of men with *BRCA1/2* alterations and measurable disease in the TRITON2 study achieved radiographic responses, which, in 60% of cases, lasted for >6 months. Fifty-two per cent also had a ≥50% decrease in PSA levels. In this study, no differences in response have been observed between patients with germline and somatic *BRCA2* alterations. In GALAHAD, the reported overall response rate for men with biallelic *BRCA1/2* alterations was 41% with a median duration of 5.5 months, and the PSA response rate was 50%. TRITON2 included patients with mono- and biallelic *BRCA1/2* alterations, while only patients with biallelic defects were eligible for GALAHAD, but in view of the reported response rates, the impact of zygosity on the response to PARPi is unclear, at least for mCRPC.

**Table 3:** Characteristics of the principal studies with PARP inhibitors in monotherapy for mCRPC.

	PROfound	TRITON2	TALAPRO-1	GALAHAD
Drug	Olaparib	Rucaparib	Talazoparib	Niraparib
	300 mg b.i.d.	600 mg b.i.d.	1 mg q.d.	300 mg q.d.
Study design	Phase 3	Phase 2	Phase 2	Phase 2
Population	mCRPC	mCRPC	mCRPC	mCRPC
	Progression to ARSi	Progression to ARSi and taxane	Progression to ARSi and taxane	Progression to ARSi and taxane
Primary objective	rPFS in pts with alterations in ATM, BRCA1, BRCA2	ORR and PSA response (≥50% decline) in pts with DDR alterations	ORR in patients with DDR alterations	ORR in patients with biallelic BRCA1/2 alterations
Specimen tested	Tumour tissue	Plasma or tumour tissue	Tumour tissue	Plasma
	Central	Central/local	Central/local	Central
Test used	FoundationOne®	FoundationOne®	FoundationOne®	Resolution-HRD®
		FoundationACT®		
		Local		
Genes screened	*ATM*, *BARD1*, *BRCA1*, *BRCA2*, *BRIP1*, *CDK12*, *CHEK1*, *CHEK2*, *FANCL*, *PALB2*, *PPP2R2A*, *RAD51B*, *RAD51C*, *RAD51D*, *RAD54L*	*ATM*, *BARD1*, *BRCA1*, *BRCA2*, *BRIP1*, *CDK12*, *CHEK2*, *FANCA*, *NBN*, *PALB2*, *RAD51*, *RAD51B*, *RAD51C*, *RAD51D*, *RAD54L*	*ATM*, *ATR*, *BRCA1*, *BRCA2*, *CHEK2*, *FANCA*, *MLH1*, *MRE11A*, *NBN*, *PALB2*, *RAD51C*	*ATM*, *BRCA1*, *BRCA2*, *BRIP1*, *CHEK2*, *FANCA*, *HDAC2*, *PALB2*
Genomic alteration required	Mono- and biallelic DDR alterations			Biallelic DDR alterations

See main text for definitions.

These findings are consistent with a report by Jonsson et al.,^22^ which showed that the clinical benefit of PARPi in *BRCA*-associated cancer types, including those with increased heritable cancer risk, such as prostate, breast, ovarian and pancreatic cancer, was similar regardless of somatic, germline and mono- or biallelic inactivation.

### DDR alterations confer different sensitivity to PARP inhibition

The benefit of PARPi in *BRCA2* carriers are well established across all the above-mentioned studies; however, it is unclear whether other DDR alterations beyond *BRCA2* may predict response to PARPi. In the TRITON study, only 15% and 10% of patients with non-*BRCA* DDR defects achieved radiographic and PSA responses, respectively.^58^ Similar response rates have been observed in GALAHAD.^57^ Despite early preclinical data suggesting that patients with *ATM* alterations would respond to PARPi,^47^ clinical evidence has proven otherwise. First, in a retrospective analysis of 23 mCRPC patients treated with olaparib, none of those with *ATM* (*n* = 6) achieved PSA responses compared with 76% of patients with *BRCA1/2* alterations (*n* = 17). Disease progression occurred significantly earlier on *ATM*-mutated patients.^59^ These findings have later been confirmed in clinical trials as *ATM*-deficient tumours have consistently shown limited response to different PARPi.^19,54,57,58^ Similar lack of response has been seen in patients with *CDK12* inactivation.^19,54,57,58^ On the contrary, promising results have been observed for patients with alterations in *PALB2*, *BRIP1*, *FANCA* or *RAD51B*.^58^ However, due to the low prevalence of these aberrations, further data are needed to fully understand their value as predictors of response to PARPi.

### PARPi and ARSi

Crosstalk between the AR and DNA repair pathways has been extensively described.^60–62^ First, PARP is involved in androgen-dependent transcription and PARPi, therefore, impair this process.^63^ Second, the AR pathway regulates the transcription of DNA repair genes; androgen depletion, therefore, impairs HRR,^60^ which might render the tumour susceptible to PARPi, regardless of HRR mutation status. A Phase 2 randomised trial assessed the efficacy and tolerability of olaparib in combination with abiraterone compared with abiraterone and placebo in mCRPC patients pretreated with docetaxel, irrespective of their DDR status. Eleven out of 71 (15%) men in the olaparib arm and ten out of 71 (14%) patients in the control arm had mutations in HRR genes. However, 61% of patients had only partially characterised HRR status, as the results of germline and plasma testing could not be confirmed by tumour analysis. Time-to-radiographic progression was significantly prolonged in the olaparib plus abiraterone group compared with the abiraterone alone group (13.8 vs. 8.2 months, *P* = 0.034), regardless of HRR status. No differences in radiological response rates or in PSA responses were observed between the two arms. Importantly, 54% of patients in the olaparib plus abiraterone arm presented severe adverse events compared with 28% in the abiraterone only group, including seven (10%) patients with a serious cardiovascular event.^64^ Supported by this early data, several currently ongoing Phase 3 trials aim to address the potential synergy between PARPi and ARSi in all mCRPC patients, irrespective of DDR status (Table 2).

### Other strategies to treat DDR-deficient tumours

Activation of the DNA damage-sensing proteins ATM and ATR stimulates different downstream effectors, such as checkpoint kinases 1 and 2 (Chk1 and Chk2, encoded by *CHEK1* and *CHEK2* genes, respectively) and Wee1, all of which are involved in the maintenance of genomic integrity^65^ (Box 1). Although previous data have suggested that ATM-deficient tumours show an increased sensitivity to PARPi,^47^ preclinical findings contradict these reports.^66^ Clinical trials conducted in mCRPC patients have shown very limited benefit of PARPi for patients with *ATM* alterations.^19,54–59^ Thus, other strategies should be explored in these patients, and several ATR, ATM and Chk inhibitors either alone or in combination with other agents are at different stages of preclinical or clinical development^67,68^ (Table 2).

While PROfound^19^ demonstrated some potential time-to-progression benefit for olaparib compared with an alternative second ARSi in *CDK12*-altered mCRPC patients, no similar benefit was observed in TOPARP-B^54^ or in TRITON2.^58^ *CDK12* has been linked to the HRR pathway, but is also a key player in maintaining genomic stability, and *CDK12* inactivation delineates a distinct subgroup of prostate cancers that are characterised by marked genomic instability with focal tandem duplications, increased levels of T cell infiltration and neoantigens.^32,69^ Thus, *CDK12*-mutated tumours might constitute a different subgroup of prostate cancer that could benefit from immunotherapy.^32,69,70^ In that respect, the largest cohort to date of *CDK12*-inactivated prostate cancer patients treated with immunotherapy has been provided by two independent retrospective multicentre series that have, together, described the outcomes of 112 *CDK12*-mutated tumours.^17,18^ Of these, 28 received diverse immunotherapy regimens showing favourable responses even in some heavily pretreated cases. Another group of tumours that may benefit from immune checkpoint inhibitors are those MMR-deficient or microsatellite instability-high (MSI-H)^71^ (Box 1). In 2017, the US Food and Drug Administration approved pembrolizumab for the treatment of solid tumours based on biomarker status (MMR-deficient or MSI-H tumours) rather than the primary site/tumour type. However, exploratory analysis of data from KEYNOTE-199, a multicohort Phase 2 trial of pembrolizumab in mCRPC patients, failed to show a clear correlation between responses to pembrolizumab and DDR defects or MMR deficiency in tumours.^72^ Further studies assessing the role of immunotherapy in prostate cancer are ongoing (NCT04104893, NCT04019964 and NCT03570619).

## Germline and somatic DDR testing in prostate cancer

As discussed above, the identification of DDR defects in prostate cancer could have prognostic and therapeutic implications. Following the approval of PARPi for the treatment of prostate cancer, genomic testing should be considered in most mCRPC patients. Some DDR alterations may occur early in the evolution of aggressive prostate tumours and could be detected in the diagnostic biopsy or prostatectomy. Some other genomic events may be acquired during the progression of the disease and metastatic tumour biopsy has been the preferred approach to collect information on advanced cancer features. However, biopsies of metastatic lesions can be challenging or not feasible, and at the same time, a single biopsy may not capture heterogeneity across metastases. Besides, the PROfound study has revealed that 30% of such samples might not be of sufficient quality for next-generation sequencing to be carried out.^20^ The analysis of circulating free DNA is a promising approach as it might overcome the difficulty in obtaining tissue in some cases, but it is still early to conclude that these assays can be used reliably. Currently, constitutive DNA testing might be more straightforward, as germline variants and mutations can be easily and reliably evaluated from peripheral blood or saliva, and accepted standards for reporting variants exist. However, somatic and germline DDR mutations may have a similar prevalence, so by screening only for the germline variants, approximately half of the patients who might benefit from PARPi would not be identified.

The high prevalence of germline DDR alterations in prostate cancer and the fact that 30% of patients harbouring a germline DDR mutation do not have a relative affected by cancer^9,13^ have led to the recommendation of germline screening for all patients with high-risk localised prostate cancer and metastatic disease.^73,74^ Unfortunately, most guidelines of clinical practice have not implemented this recommendation, and most patients with advanced disease are not offered germline screening. According to the current guidelines, however, families of prostate cancer patients found to carry a germline *BRCA2* mutation would not fulfil the criteria for genetic testing until a median of two further breast and/or ovarian cancer cases per family occur.^75^ The identification of an inherited mutation in a prostate cancer patient would not only have implications for the patient, but should also be followed by genetic testing in all related family members, providing the opportunity for early cancer-specific screening and risk reduction strategies in those found to be carriers. Unaffected men >40 years found to carry a *BRCA2* mutation are recommended to have annual PSA-based prostate cancer screening.^24^ Recommendations for male carriers of germline mutations in non-*BRCA* genes are less clear. Protocols for prevention and early detection of other tumour types are well established for individuals carrying mutations in certain DDR genes, but this is beyond the scope of this review.

## Conclusions and future directions

A sizeable number of patients with prostate cancer have defects in genes that are involved in the DDR pathway. Such alterations are significantly more prevalent in metastatic than in localised prostate cancer. While some DDR alterations may be acquired with time or in response to therapies, others are probably early events in the evolution of aggressive prostate tumours. A significant proportion of the alterations detected in the tumour are already present in the germline. Identification of these inherited alterations is relevant as it should prompt cascade testing in the relatives. Germline mutation carriers are at risk of different cancer types (i.e. breast, ovarian, prostate, colorectal, etc.) and may benefit from screening and early detection programmes. *BRCA2* is the most frequently altered DDR gene, both in the germline and somatic. Germline *BRCA2* mutations have consistently been associated with poor outcomes in prostate cancer at different disease stages, but the clinical impact of somatic *BRCA2* alterations is unclear. *CDK12* inactivation also seems to correlate with aggressive disease, but further research is needed to understand the clinical implications of most somatic and germline DDR alterations and the most appropriate management of these patients.

Some alterations in DDR genes, particularly in those involved in HRR, are predictors of response to PARP inhibition. Somatic, germline and bi- and monoallellic alterations in *BRCA1* and *BRCA2* have been associated with response to PARPi in clinical trials. Whether there is a differential response based on the alteration type is yet to be clarified. Innate resistance in *BRCA1/2*-altered patients without previous exposition to platinum-based chemotherapy or PARPi has also been noted. Patients with *ATM* and *CDK12* alterations seem to get little advantage from the treatment with PARPi in monotherapy, although several trials are ongoing to address other therapeutic strategies involving *ATR* inhibitors and immunotherapy regimens among others. It is unclear whether less prevalent genomic events predict response to PARPi, although promising antitumor activity has been seen in patients with *PALB2*, *BRIP1* or *FANCA* aberrations. Furthermore, responses to PARPi have been reported in biomarker-negative patients, suggesting that there may be DDR dysfunctions undetected by next-generation sequencing that reflect the multiple elements of this complex pathway.^76–78^ Further clinical trials implementing functional assays would be needed to optimise patient selection for PARPi treatments.

To date, clinical trials with PARPi have focused in the mCRPC setting; however, these drugs may also result in a clinical benefit if used in HPSC stages as addressed by several ongoing or planned studies. Usually, these trials involve the combination of a PARPi with continuous ADT or ADT plus ARSi, but an attractive hypothesis tested in the NCT03047135 study is that PARPi in monotherapy (without any antiandrogen therapies) may be active and safe in selected patients with biochemical relapse after prostatectomy preventing these men from the side effects related to androgen depletion. Another important area of research is the synergy between AR and DDR pathways and the potential benefit of combining ARSi and PARPi to treat mCRPC or mHPSC patients unselected for DDR alterations.

PARPi are the first targeted therapy approved for men with advanced prostate cancer, but other targeted therapies have shown promising results and may also be available in a short time. This is a paradigm shift in the management of prostate cancer that has finally entered the era of precision medicine. However, implementing these therapies in daily clinical practice does not come without challenges. First, it may require a level of knowledge about genomics and genetics that may exceed what most physicians received during training. Scientific societies should provide educational support to maximise the benefits of these therapies to patients. Second, acquisition of tissue for the identification of DDR defects and other targets may in some cases be a major difficulty. As previously discussed, there are molecular events that can be identified in the diagnostic biopsy, but this may have been taken years before the patient develops mCRPC, and therefore untraceable. Metastatic tumour biopsies are the preferred approach to obtain information regarding mCRPC tumour features, but these are not always feasible. Besides, a third of samples analysed in the context of clinical trials were inadequate for sequencing using some of the currently available commercial tests. Germline samples are easily collected and analysed, but limiting DDR testing just to germline alterations, about half of the patients with DDR defects (somatic) would not be detected. The development of a liquid biopsy approach may overcome these challenges providing non-invasive tumour samples that could also be used to monitor the emergence of secondary mutations that may restore the function of the gene previously altered.^79,80^

Despite the limitations of the studies reported to date, the evidence gaps and the difficulties to identify all patients that may benefit from these therapies, PARPi have finally brought precision oncology to prostate cancer, opening the door to new and exciting times.

## Data Availability

Not applicable.
